# Potential Risk of Asymptomatic Osteomyelitis around Mandibular Third Molar Tooth for Aged People: A Computed Tomography and Histopathologic Study

**DOI:** 10.1371/journal.pone.0073897

**Published:** 2013-09-10

**Authors:** Ikuya Miyamoto, Ayataka Ishikawa, Yasuhiro Morimoto, Tetsu Takahashi

**Affiliations:** 1 Division of Oral Medicine, Kyushu Dental University, Fukuoka, Japan; 2 Department of Pathology, Saitama Cancer Center, Saitama, Japan; 3 Division of Oral and Maxillofacial Radiology, Kyushu Dental University, Fukuoka, Japan; 4 Division of Oral and Maxillofacial Surgery, Department of Oral Medicine and Surgery, Tohoku University Graduate School of Dentistry, Sendai, Japan; University of Pittsburgh, United States of America

## Abstract

The purpose of this study was to explore the relationship between bone mineral density and histopathological features of mandibular alveolar bone evaluated quantitatively by Hounsfield units [HU] and by histopathology in human subjects. Fifty-six mandibular molars were extracted in 50 patients. Computed tomography was obtained preoperatively, and a cortical bone biopsy was obtained on the extracted sites for histopathological evaluation. The mean cortical and cancellous bone radiodensity was 1846±118 HU and 926±436 HU, respectively. There was no correlation between age and cortical bone HU (r = −0.004, *P = *0.976); however, the correlation between age and cancellous bone HU was significant (r = 0.574, *P*<0.0000). Significant differences in the cancellous bone between young (0–30 years), middle (31–60 years) and old patient groups (61< years) were evident (*P*<0.05), whereas the cortical bone presented no significant differences. The histopathological evaluation showed that the young patient group had relatively few osteomyelitis, whereas the old patient group showed 100% focal sclerotic osteomyelitis regardless of the fact that the patients had no clinical symptoms. The mean osteocyte number/unit bone area was 170.7±82.2. Negative correlation between age and osteocyte number was significant (r = −0.51, *P*<0.0001). Mean lacunae numbers/unit cortical bone area were 413.1±130 with non-significant negative correlation (r = −0.257, P = 0.056). The mean empty lacunae numbers/cortical bone were 242.5±145, with no correlation (r = 0.081, P = 0.559). The young patients had high osteocyte number, whereas the old patients showed reduction of the osteocytes in the cortical bone (*P*<0.05). Bone quality might correlate better to viable cell numbers, which influenced the osseous healing. It is suggested that the outermost layer of cortical bone may have lost its cellular activities over the years due to chronic infection, which may have provoked sclerotic changes in the cancellous bone around tooth.

## Introduction

Reportedly, age is a consistent factor in the determination of surgical difficulty of the third molar teeth extraction considering the differences in bone density associated with age [1]–[3]. As patient ages, the alveolar bone around the teeth becomes highly calcified, therefore is less elastic and is less likely to bend under the forces of tooth extraction. Similarly, the osseous healing is less favorable with more postoperative sequelae [4].

Bone quality is an important factor in the success of dental surgery [5]. Although there is no consensus regarding the definition of bone quality, factors such as bone mineral density (BMD), cortical bone thickness, and trabecular density have been suggested to be an important factor [6]. Lekholm and Zarb proposed a jaw bone classification in which the quality is rated from 1 to 4, depending on the amount of compact and cancellous bone present [7]. Good bone quality is exhibited by relatively thick cortical bone, which is advantageous for the initial stabilization of dental implant placement. For this reason, preoperative examination of the host bone is important for treatment predictability.

Computed tomography (CT) is currently the only diagnostic imaging technique that allows for a rough determination of the structure and density of the jaw bone [8], [9]. It is also an excellent tool for assessing the relative distribution of cortical and cancellous bone [10], [11]. In the absence of a clear definition of bone quality, a more practical definition might be to rate the bone hardness, which is experienced during osteotomy [12]. In our previous study, cortical bone thickness assessment using CT and biomechanical data were suggested to be a significant factor for initial implant stabilization [13]. It can be said that the status of the cortical alveolar bone is crucial for clinical treatment success.

Osteoporosis is a multifactorial, age-related metabolic bone disease characterized by low BMD [14]. The definition of bone quality used in the field of osteoporosis is not easily converted into a definite parameter. It has been related to the mechanical properties of the bone, and especially to its strength and stiffness, which are likely to be influenced by external and internal shape and size, as well as by the biomechanical properties of the material within [14]. Although the BMD of the bone and the radiodensity (measured in Hounsfield units, HU) have shown correlation, there is no clear evidence that bone quality correlates with HU [15], [16]. Accordingly, there may be another factor that regulates the so-called bone quality.

The purpose of this study was to explore the relationship between bone mineral density and histopathological features of the mandibular alveolar bone by means of bone quality measured by HU and histopathology in human subjects.

## Materials and Methods

### Study Participants and Extraction Sites

Inclusion criteria for this study included: erupted or impacted third molar teeth or a supernumerary tooth at the third molar region with no associated pathology, no medical conditions or medications that might alter bone condition, and the patients had to be categorized as Physical Status I or II according to the American Society of Anesthesiologists Physical Classification System [17]. Patients were excluded from the study if they had a history of previous or present radiotherapy in the third molar tooth region of the lower jaw, or were on long-term corticosteroid or bisphosphonate medication, or had a systemic bone disorder. A total 50 patients participated in this study (23 male, 27 female; age range 9–83; mean age 43.6 years). At 56 mandibular alveolar ridge sites, third molar tooth and supernumerary tooth extraction was performed. After full explanation of the study, written informed consent was obtained from all participants. In case of patients under the age of 20 years, a written informed consent was obtained from both the parents and the patients. All clinical and biological samples were collected following patient consent. The ethical committee of Kyushu Dental University approved the protocol (2012: 23–43).

### CT and Bone Density Measurements

**Figure 1:**
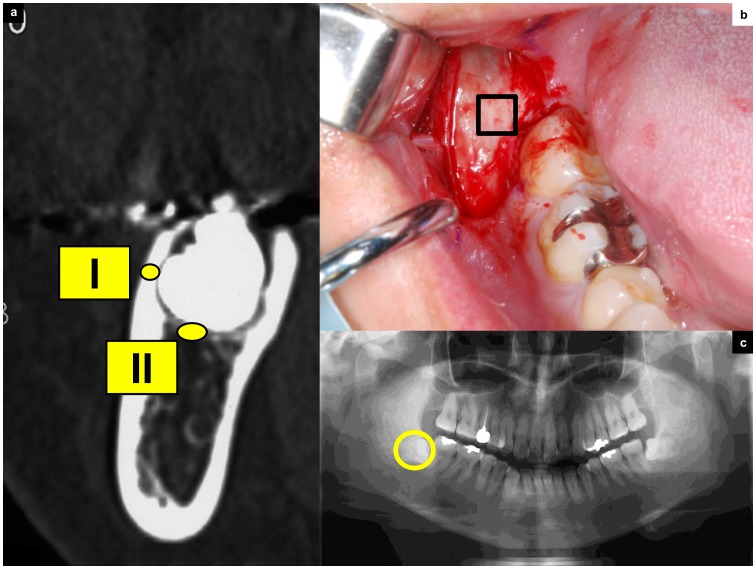
Design of the tooth extraction procedure and CT measurement. (a) This CT image details the condition of third molar teeth covered with cortical bone. The regions of interest in CT image analysis were (I) the buccal cortical bone area, which was taken for biopsy during surgery; and (II) the region under the cortical bone, which indicates cancellous bone near the third molar tooth. (b) Removal of the third molar tooth. The envelope flap is raised, revealing the cortical bone. The line indicates the biopsy area. (c) Orthopantomographs showing impacted third molar teeth.

Computed tomography (CT) was employed for the preoperative evaluation of the jaw bone, using a Toshiba X Vision RE™ machine with a single row of detectors (Toshiba Co. Ltd., Tokyo, Japan). The CT images for all patients were obtained with the occlusal plane perpendicular to the ground, and were obtained in a helical manner with contiguous sections 2 mm thick. The images were photographed with bone-tissue windows using a 400-Hounsfield units (HU) window level and a 2000-HU window width. The HU measurements were performed at two sites. The HU in the bone at the buccal cortical area of the mandibular third molar were investigated on the monitor using the software associated with the CT scanning system. The region of interest regarding the HU was commonly cut down for the third molar extraction to clarify the crown. At the same time, the radiodensity of the cancellous bone under the cortical area was measured in HU (Figure 1a).

The interobserver reliability of HU measurements was calculated by the intraclass correlation coefficient (ICC) by 2 observers (IM and YM). Two weeks later, one of the observers repeated the above radiographic assessments to determine the intraobserver reliability. Each examiner was blinded to the other measurements and the patient data. The order of the measurements was assigned randomly to each observer.

### Bone Biopsy

A bone fragment (5 × 5 mm) including the outer cortex was excised on the buccal side of the impacted third molar. The thickness of this zone was assessed using preoperative CT. All specimens were biopsied by a trained oral surgeon at the time of surgery (Figure 1b, c). The cancellous bone located under the coronal part of the impacted teeth was not taken for biopsy from an ethical standpoint since standard tooth extraction procedure does not remove this area of alveolar bone.

The samples were fixed in 10% neural buffered formalin for 12 h, fixed in 70% ethanol, dehydrated and decalcified in formic acid for 48 h, dehydrated in a graded ethanol series, and embedded in paraffin. A series of 4-µm sections were cut using a Polycut E microtome and stained with hematoxylin-eosin. For each biopsy specimen, approximately 13–15 longitudinal sections were used for light microscopic examination under an Olympus BX51 microscope (Olympus Optical Co., Tokyo, Japan).

### Bone Pathology

**Figure 2:**
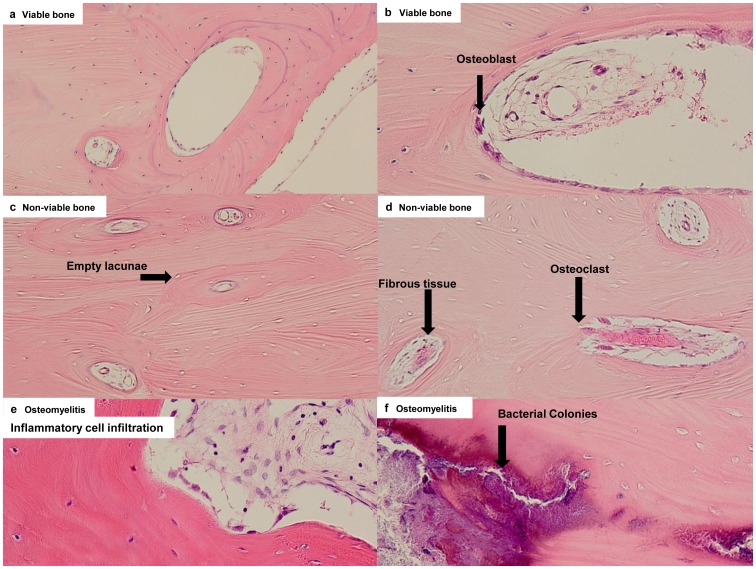
Pathological features. (a) A representative image of viable bone (x100). Normal-appearing bone that is remodeling, with osteoblasts, and osteocytes present. Occasional empty osteocytic lacunae (<10–20% of lacunae per high magnification) may be present in the absence of an inflammatory reaction. (b) High magnification of viable bone (x200). There are osteoblasts at the border of bone marrow. Arrow head shows osteoblasts. (c) Non-viable bone (×100). Empty osteocytic lacunae and absence of osteoblasts. Empty lacunae are representative of osteocytic death. Arrow head shows empty lacunae. (d) High magnification of non-viable bone (×200). Osteoclasts are present; however, the inflammatory reaction is minimal. (e) Osteomyelitis: prominent inflammatory cell infiltration in fibrous marrow, with osteoblastic activity creating irregular bony trabeculae (×200). (f) High magnification of osteomyelitis (×200). Necrotic bone (sequestrum), abundant bacterial colonies are shown.

Bone pathological diagnosis was based on the criteria reported by Kassolis et al. [18]. Figure 2 depicts typical histopathological images of the respective specimens (Figure 2a–e).

In brief, the criterion were as follows:

- *Viable bone:* visibly normal bone that is remodeling, with osteoblasts, and osteocytes present. Occasional empty osteocytic lacunae (<10–20% of lacunae per high magnification) may be present in the absence of an inflammatory reaction.
- *Non-viable bone:* presence of empty osteocytic lacunae and absence of osteoblasts (more than 80% of lacunae/high magnification) and fatty marrow. Osteoclasts are often present; however, the inflammatory reaction is minimal, if present.
- *Osteomyelitis:* prominent inflammatory cell infiltration in the fibrous marrow, with osteoblastic activity creating irregular bony trabeculae. Necrotic bone (sequestrum) and bacterial colonies are often present. Acute or chronic designations are used to identify the specimens with inflammatory cell infiltrate, characterized by either polymorphonuclear leukocytes or plasma cells and lymphocytes, respectively.

### Histomorphometric Analysis

Histomorphometric measurements were performed on three fields of each section with a ×20 objective and a ×10 eyepiece. Three fields were randomly selected for evaluation in each tissue specimen. Total 168 (56 samples × 3 times) measurements were performed in this study. A mean value was determined for each specimen. In the current study, the osteocyte population was determined by evaluating the density of osteocytes that reflected the characteristics of the osteocytic network. Similarly, empty lacunae were considered representative of osteocytic death. The osteocytic density (osteocyte number/cortical bone area, cells/mm^2^), density of lacunae (total number of lacunae/cortical bone area, lacunae/mm^2^), and density of empty lacunae (number of empty lacunae/cortical bone, empty lacunae/mm^2^) were each measured according to previous studies [19], [20]. All specimens were obtained by a licensed oral pathology service at Kyushu Dental University, which provided access to the slides and reports.

### Statistical Analysis

Data are presented as mean ± standard deviation unless otherwise noted. The data were entered into a personal computer and analyzed using JMP Software for Windows® (version 5.1, SAS Institute Inc., Cary, NC). One-way analysis of variance (ANOVA) was used to examine the significance of the differences in aging between each of the pathological features. For post-hoc multiple-comparison procedures, we used the Bonferroni correction, which set the level of significance at 0.05 per number of comparisons. The relationship between cortical and cancellous bone radiodensity (measured in HU), as well as the correlation between osteocyte number, total lacunae number, and empty lacunae number, were examined using Pearson’s correlation coefficient. A *P* value of less than 0.05 was considered statistically significant. For reliability testing, ICC was used for continuous variables. To determine the reliability of the cortical and the cancellous HU value (continuous variable), the ICC and their 95% CIs were used to summarize the intra- and interobserver reliability [21].

## Results

### Age-related Changes in CT Imaging

**Figure 3:**
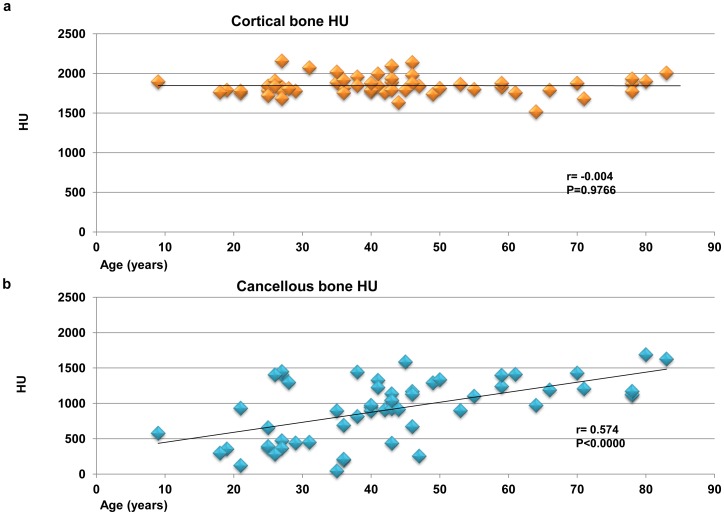
Correlation between HU and age in cortical and cancellous bone. (a) Cortical bone radiodensity is not significantly associated with age (n = 56, r = −0.004, *P = *0.977). (b) There is a statistically significant positive linear correlation between cancellous bone radiodensity and age (n = 56, r = 0.574, *P*<0.0000).

The radiodensity showed favorable reliability in terms of the ICC values, which supported the reliability of the HU measurement (Table 1). The mean cortical bone radiodensity was 1846±118 HU, and the mean cancellous bone radiodensity was 926±436 HU. There was no significant correlation between age and the radiodensity of cortical mandibular bone (r = −0.004, *P = *0.976; Figure 3a). However, the correlation between age and the radiodensity in mandibular cancellous bone was significant (r = 0.574, *P*<0.0000; Figure 3b).

**Table 1:** Inter- and intraobserver reliability of the HU measurement.

	Intraclass correlation coefficient	95% Confidence interval
**Interobserver reliability**	0.88	0.82–0.93
**Intraobserver reliability**	0.96	0.93–0.98

**Figure 4:**
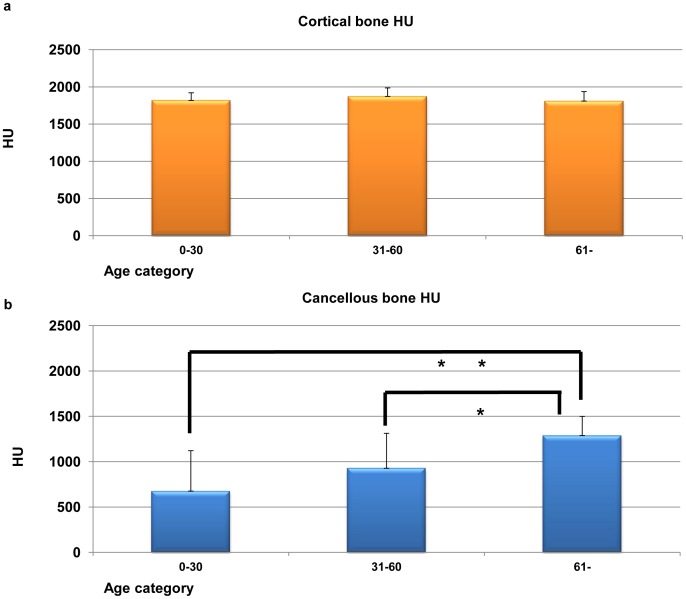
Three age categories of cortical and cancellous bone radiodensity. (a) Three age categories of cortical bone radiodensity are not significantly differences (ANOVA and Bonferroni correction, *P* = 0.180; Figure 3c). (b) Three age categories of cancellous bone radiodensity are significant differences (ANOVA and Bonferroni correction, *P<0.05, **P<0.01; Figure 3d).

Three age categories of bone radiodensity were shown in Figures 4a and 4b. For the young generation (0–30 year old), cortical bone radiodensity was 1819±103 HU. The middle age group (31–60 year old) was 1874±112 HU, and the old generation (61< year old) was 1811±127 HU. There were no statistically differences (ANOVA and Bonferroni correction, P = 0.180). On the other hand, the radiodensity of the cancellous bone in the young generation was 675±447 HU, the middle age group was 927±386 HU and the old generation was 1288±212 HU. One-way analysis of variance, which was used to compare bone radiodensity in three age categories, demonstrated significant differences between each groups of cancellous bone (ANOVA and Bonferroni correction, *P<0.05, **P<0.01; Figures 4a and 4b).

### Pathological Features

**Figure 5:**
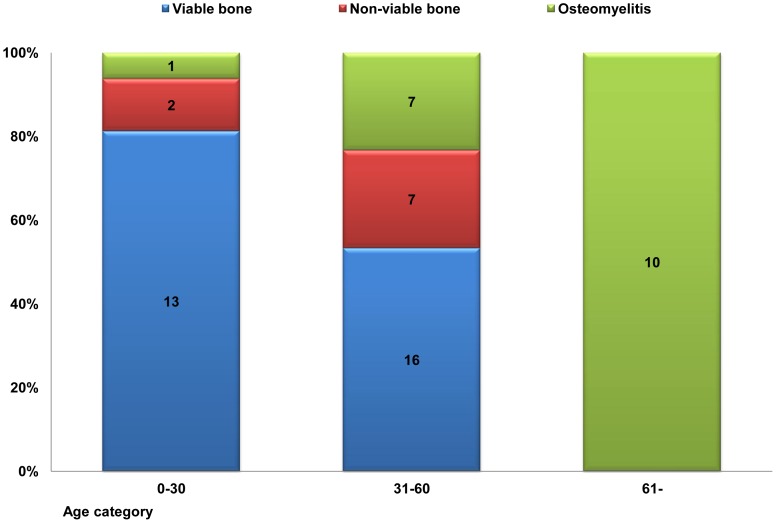
The distribution of three age categories of pathological features. The distribution of three age categories of pathological features. Old generation shows 100% focal sclerotic osteomyelitis histopathologically, whereas young generation shows relatively few osteomyelitis.

The diagnostic pathology reports for 56 alveolar cortical bone specimens indicated that 29 were viable bone (51.8%); 9 were non-viable bone (16.1%); and 18 had osteomyelitis (32.1%). The distributions of three age categories of the pathological features are shown in Figure 5. The young generation specimens indicated that 13 were viable bone (81%); 2 were non-viable bone (13%); and 1 had osteomyelitis (6%). The middle age group showed that 16 were viable bone (53%); 7 were non-viable bone (23%); and 7 had osteomyelitis (23%), and the old generation showed that all samples had osteomyelitis (100%).

### Histomorphometry

**Figure 6:**
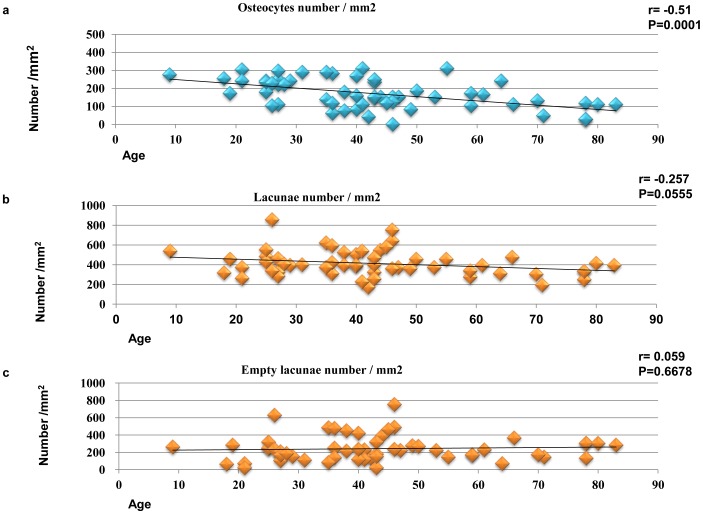
Correlation between histological features and age in cortical bone. (a) There is a statistically significant negative correlation between age and osteocytic density (cells/mm^2^) (n = 56, r = −0.51, *P = *0.0001). (b) Density of bone lacunae (lacunae/mm^2^) does not correlate significantly with age (n = 56, r = −0.257, *P = *0.0555). (c) Density of empty lacunae (empty lacunae/mm^2^) does not correlate significantly with age (n = 56, r = 0.059, *P = *0.6678).

Histomorphometric analysis of the osteocytic density in the cortical bone surrounding the third molar tooth revealed age-dependent relations. The mean osteocytic density was 170.7±82.2 cells/mm^2^. The correlation between age and osteocytic density in mandibular cortical bone was statistically significant (r = −0.51, *P*<0.0001; Figure 6a). The mean density of lacunae was 413.1±130 lacunae/mm^2^. Although there was a weak negative correlation between age and the density of lacunae, it was not statistically significant (r = −0.257, *P* = 0.056; Figure 6b). The mean density of the empty lacunae was 242.5±146 empty lacunae/mm^2^. There was no correlation between age and the density of the empty lacunae (r = 0.059, *P* = 0.668; Figure 6c).

**Figure 7:**
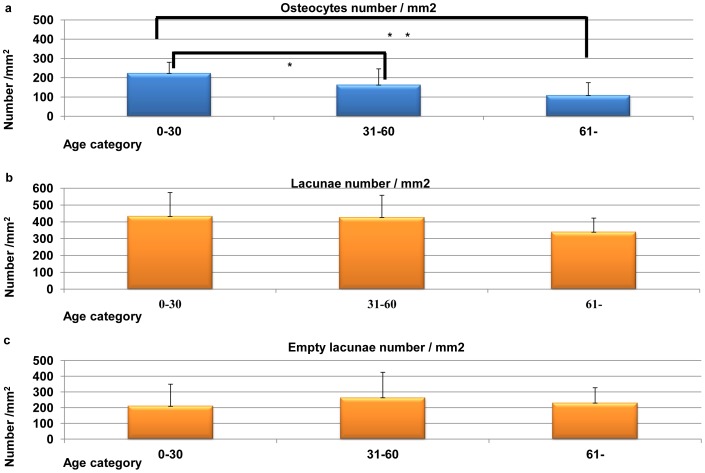
Three age categories of histological features of cortical bone. (a) Three age categories of osteocytic density (cells/mm^2^). There are statistically significant differences (ANOVA and Bonferroni correction, *P<0.05, **P<0.01). (b) Three age categories of bone lacunae density (lacunae/mm^2^). There are no statistically significant differences (ANOVA and Bonferroni correction, *P* = 0.139). (c) Three age categories of empty bone lacunae density (empty lacunae/mm^2^). There are no statistically significant differences (ANOVA and Bonferroni correction, *P* = 0.470).

The osteocyte numbers for all age categories are shown in Figure 7a. In brief, the young generation had 223.4±57.2 cells/mm^2^, the middle age group had 163.1±83.1 cells/mm, and the old generation was 109.0±65.2 cells/mm^2^. There were statistically significant differences between each cancellous bone groups (ANOVA and Bonferroni correction, *P<0.05, **P<0.01; Figure 7a). The mean lacunae densities for the three age categories are shown in Figure 7b. The young generation had 433.0±140 lacunae/mm^2^, the middle age group had 427.1±131 lacunae/mm^2^, and the old generation had 339.5±83.0 lacunae/mm^2^. There were no statistically significant differences (*P* = 0.139). The mean density of the empty lacunae for the three age categories are shown in Figure 7c. The young generation had 209.6±140 empty lacunae/mm^2^, the middle age group had 264.0±161 empty lacunae/mm^2^, and the old generation had 230.5±96.4 empty lacunae/mm^2^. There were no statistically significant differences (*P* = 0.470).

## Discussion

In the present study, the characteristics of the bone surrounding the third molar tooth were investigated quantitatively with both CT imaging and histopathology. In the outermost layer of the cortical bone, the osteocyte density and the density of the bone lacunae had decreased. In contrast, it seemed that the endosteum formed new bone into the bone marrow for the cancellous bone under the tooth, which, led to an increase of the BMD measured by the CT imaging. Based in the histopathological findings, the density of the lacunae decreased in the cortical bone with cellular damages and octeocyte death, which resulted in mineralization of the lacunae, better known as micropetrosis [22]–[26]. Micropetrosis presents an increase of the radiodensity in the cortical bone, however, from the results obtained in the current study, the CT imaging could not detect the differences. These findings strongly suggests that the alveolar bone remodeling in the jaw bone may be part an infection-related phenomenon, possibly due to sclerotic changes in the cancellous bone and decreased the cortical osteocytes numbers. This biologic phenomenon is especially a notable characteristic, which distinguishes the mandible from other long bone. Few published studies address aging and the jawbone with regard to bone quality, although there are several reports concerning the long bones [27].

Osteocytes are located in the ellipsoid cavities (lacunae) and are extensively interlinked to one another, as well as to the bone-forming cells, or osteoblasts, by cytoplasmic processes inside the canaliculi [27]–[29]. The osteocytic network is believed to sense the local environment in bone, and to affect bone formation (osteoblasts) and resorption (osteoclasts) [30]. The results of the current study demonstrated sclerotic bone changes, which is contradictory to the known phenomena in the long bone. With stimulation, the porosity of the outer layer of long bone increases, the inner layer of cortical bone decreases, and trabecular bone decreases. This difference between the mandible and the long bone might originate from stimulation and direction. With an anatomical reason in the mandible, teeth are directed towards the bone marrow, and chronic bacterial infection is transferred in the direction of the bone marrow via the periodontal ligament [31]. Conversely, in the long bone, the main stimulation to the bone is mechanical loading, and it primarily stimulates the periosteum. Clinical observation of edentulous patients after implant insertion reported increased bone volume in the jaw after functional prosthesis loading [32]. These results might suggest that mechanical loading under physiologically optimal loading conditions could result in the formation of new bone due to periosteum stimulation. In contrast, stimulation of the inner bone marrow may activate endosteum bone apposition.

It is now accepted that one role of bone remodeling is to repair microdamage accumulated in long bone [19], [33]. This microcrack phenomenon is also associated with a decrease in cortical osteocyte lacunar density [34]–[37]. It has been suggested that microdamage accumulation and the failure to repair properly may cause an increase in the percentage of poor quality bone and delay wound healing [19]–[20], [27].

In the jaw bone, and particularly in the third molar area, the decrease in the number of osteocytes and lacunae may be a result of the damage response against bacterial infection (from intraoral microorganisms to the outer cortical bone) than microdamage, which is related to load transmission. In this study, there was no clear evidence of microcracks in these decalcified specimens. Future investigation of undecalcified specimens is needed to detect microdamage in the mandible.

A number of authors have reported that age is a consistent factor in the determination of surgical difficulty at the removal of third molar teeth, considering the differences in bone density associated with age [1]–[4]. The positive correlation may be related to an increase in bone density, which may require more handling during the operation. It can be easily speculated that the sclerotic bone might make removal of the third molar teeth more difficult. Moreover, the low density of osteocytes and reduced vascularity might negatively influence postoperative bone healing. From the obtained results, it can be suggested that considerable pathologically osteomyelitis condition around impacted tooth exist in the aged patients. Osteomyelitis of the jaw could be considered as an inflammatory condition of the bone, beginning in the medullar cavity and harvasian systems and expanding to involve the periosteum of the affected area [38]. It has been known that acute and chronic osteomyelitis of the jaw is caused mostly by a bacterial focus (odontgenic disease, pulpal and periodontal infection, extraction wounds, foreign bodies, and infected fractures) [38]. In this study, these clinical conditions were representing chronic non-suppurative inflammation, which were pathologically chronic focal sclerotic osteomyelitis of the jaw bone.

Possible mechanisms of these pathological changes are that viable bone would be damaged and transform to a non-viable bone condition with chronic bacterial infection. Osteocyte death could induce micropetrosis in the lacunae and these tissue reaction cascades induce micro bone consolidation. However, the density of the empty lacunae did not reflect its numbers particularly in osteomyelitis samples as shown in Figure 6c. This reflects reactive osteogenesis due to osteomyelitis induced by bacterial infection and these pathological conditions might increase apparent bone lacunae with inflammatory cell infiltration. Further, bacterial infection could result in sclerotic osteomyelitis with bone consolidation and reducing osteocytes cell numbers.

**Figure 8:**
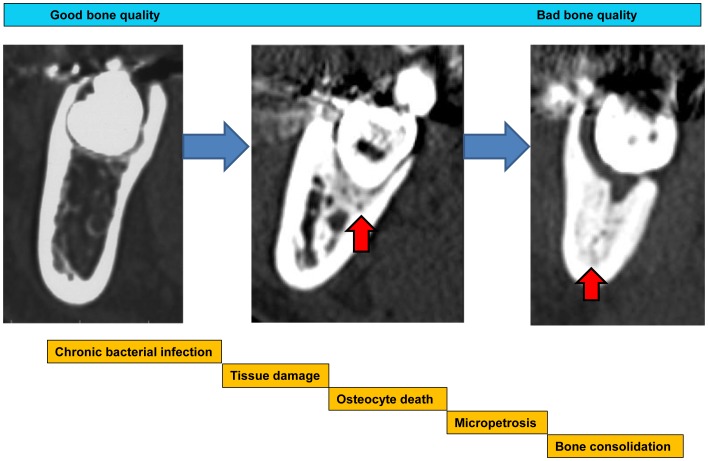
Schematic illustration of the formation mechanisms of the chronic focal bone consolidation. Schematic illustration of the formation mechanisms of the chronic focal osteomyelitis. The chronic bacterial infection around tooth would induce tissue damage and it resulted in osteocytes death and resulted in micropetrosis. These accumulation of micropetrosis might induce bone sclerosis. Red arrow head shows cancellous bone consolidation.

Conclusively, with an apparent low-grade bacterial infection, the alveolar bone around third molar tooth demonstrated sclerotic change of the cancellous bone and considerable death of the osteocyte in the cortical bone with micropetrosis evaluated by CT imaging and histopathology (Figure 8). It can be suggested that the bone quality is not solely indicated by refraction of the mineralized tissue, but might be related to the viable cellular activity and influence the healing process of bony wounds. The bone which contains enough viable cells is thought to present good bone quality and the bone with reduced cell numbers might be considered ‘bad’.

Within the limitation of the current study, it can be suggested that at the time of third molar tooth extraction, if the alveolar bone shows sclerotic change in an aged population, the removal of impacted teeth should not be considered as simple tooth extraction, but it must be regarded as treatment of sclerotic osteomyelitis around the teeth.
